# Supplementary material for: Time‑resolved multi-omic analysis of paclitaxel exposure in human iPSC‑derived sensory neurons unveils mechanisms of chemotherapy‑induced peripheral neuropathy
Source: Cell Death Dis. 2026 Feb 10;17(1):211. doi: 10.1038/s41419-026-08445-2 (PMC12921266; doi:10.1038/s41419-026-08445-2)
Supplement: Supplementary file 15 — Dataset 1. [file 41419_2026_8445_MOESM15_ESM.zip › CDD_Open_Data/04_RNA_Seq/Timeline_panel_plots_etc_2023-01-23_1609.zip!/Panel_Plots_etc/05_tmod_panel_plot_msigdb_hallmark_pval_filter_0.01_pval0.05_AUC0.5_L2FC0.5.pdf]

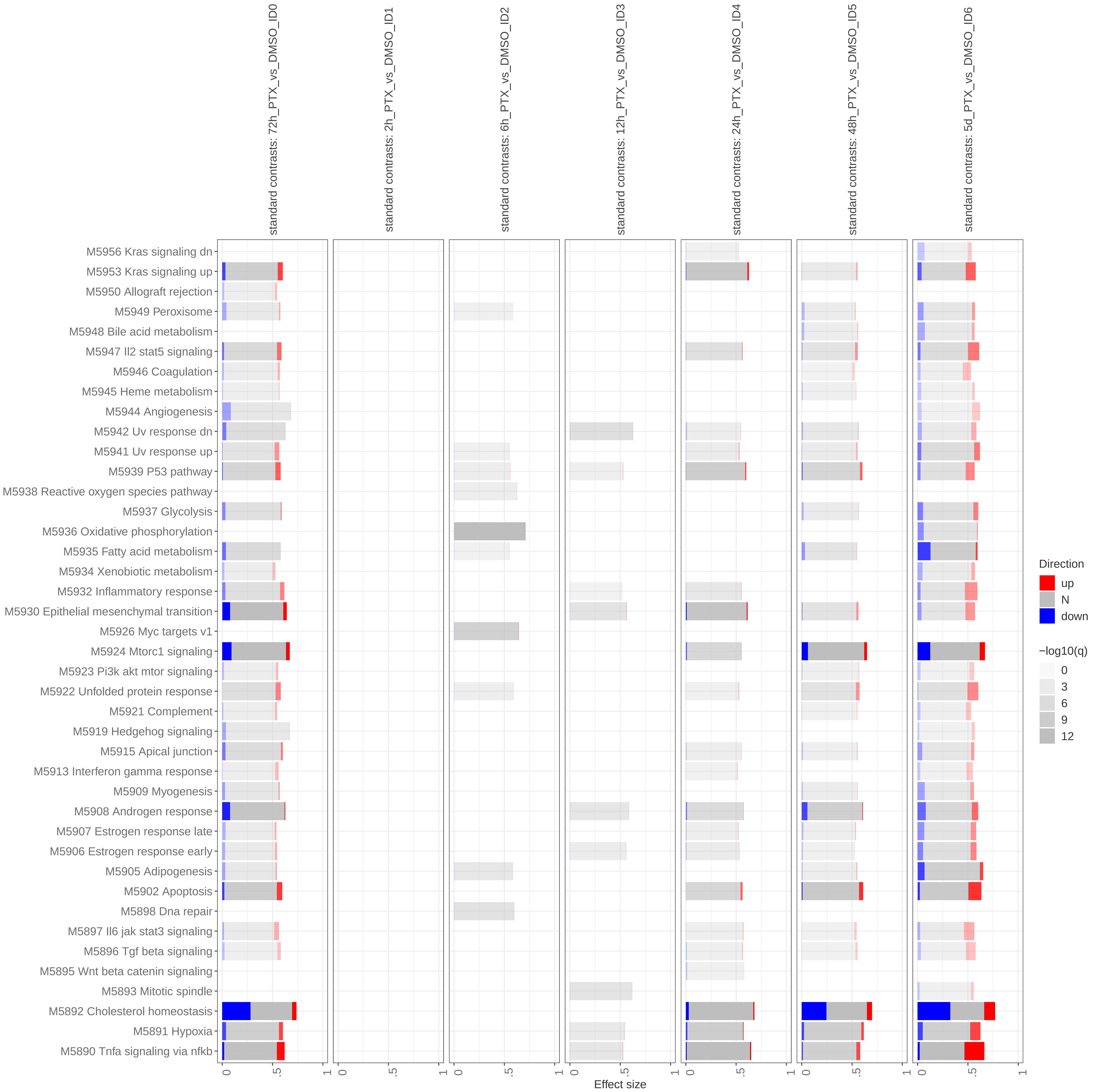

standard contrasts: 72h_PTX_vs_DMSO_ID0
standard contrasts: 2h_PTX_vs_DMSO_ID1
standard contrasts: 6h_PTX_vs_DMSO_ID2
standard contrasts: 12h_PTX_vs_DMSO_ID3
standard contrasts: 24h_PTX_vs_DMSO_ID4
standard contrasts: 48h_PTX_vs_DMSO_ID5
standard contrasts: 5d_PTX_vs_DMSO_ID6
M5956 Kras signaling dn
M5953 Kras signaling up
M5950 Allograft rejection
M5949 Peroxisome
M5948 Bile acid metabolism
M5947 Il2 stat5 signaling
M5946 Coagulation
M5945 Heme metabolism
M5944 Angiogenesis
M5942 Uv response dn
M5941 Uv response up
M5939 P53 pathway
M5938 Reactive oxygen species pathway
M5937 Glycolysis
M5936 Oxidative phosphorylation
M5935 Fatty acid metabolism
M5934 Xenobiotic metabolism
M5932 Inflammatory response
M5930 Epithelial mesenchymal transition
M5926 Myc targets v1
M5924 Mtorc1 signaling
M5923 Pi3k akt mtor signaling
M5922 Unfolded protein response
M5921 Complement
M5919 Hedgehog signaling
M5915 Apical junction
M5913 Interferon gamma response
M5909 Myogenesis
M5908 Androgen response
M5907 Estrogen response late
M5906 Estrogen response early
M5905 Adipogenesis
M5902 Apoptosis
M5898 Dna repair
M5897 Il6 jak stat3 signaling
M5896 Tgf beta signaling
M5895 Wnt beta catenin signaling
M5893 Mitotic spindle
M5892 Cholesterol homeostasis
M5891 Hypoxia
M5890 Tnfa signaling via nfkb
0
.5
1
Effect size
Direction
up
N
down
−log10(q)
0
3
6
9
12
